# A practical Java tool for small-molecule compound appraisal

**DOI:** 10.1186/s13321-015-0079-1

**Published:** 2015-06-16

**Authors:** Parisa Amani, Todd Sneyd, Sarah Preston, Neil D Young, Lyndel Mason, Ulla-Maja Bailey, Jonathan Baell, David Camp, Robin B Gasser, Alain-Dominique Gorse, Paul Taylor, Andreas Hofmann

**Affiliations:** Structural Chemistry Program, Eskitis Institute, Griffith University, Nathan, QLD Australia; Faculty of Veterinary and Agricultural Sciences, The University of Melbourne, Parkville, VIC Australia; Medicinal Chemistry, Monash Institute of Pharmaceutical Sciences (MIPS), Monash University, Parkville, VIC Australia; Griffith School of Environment, Griffith University, Nathan, QLD Australia; Queensland Facility for Advanced Bioinformatics, Institute for Molecular Bioscience, University of Queensland, St Lucia, QLD Australia; School of Biological Sciences, The University of Edinburgh, Edinburgh, Scotland, UK

**Keywords:** Compound appraisal, Molecular properties, Personal database

## Abstract

**Background:**

The increased use of small-molecule compound screening by new users from a variety of different academic backgrounds calls for adequate software to administer, appraise, analyse and exchange information obtained from screening experiments. While software and spreadsheet solutions exist, there is a need for software that can be easily deployed and is convenient to use.

**Results:**

The Java application cApp addresses this need and aids in the handling and storage of information on small-molecule compounds. The software is intended for the appraisal of compounds with respect to their physico-chemical properties, analysis in relation to adherence to likeness rules as well as recognition of pan-assay interference components and cross-linking with identical entries in the PubChem Compound Database. Results are displayed in a tabular form in a graphical interface, but can also be written in an HTML or PDF format. The output of data in ASCII format allows for further processing of data using other suitable programs. Other features include similarity searches against user-provided compound libraries and the PubChem Compound Database, as well as compound clustering based on a MaxMin algorithm.

**Conclusions:**

cApp is a personal database solution for small-molecule compounds which can handle all major chemical formats. Being a standalone software, it has no other dependency than the Java virtual machine and is thus conveniently deployed. It streamlines the analysis of molecules with respect to physico-chemical properties and drug discovery criteria; cApp is distributed under the GNU Affero General Public License version 3 and available from http://www.structuralchemistry.org/pcsb/. To download cApp, users will be asked for their name, institution and email address. A detailed manual can also be downloaded from this site, and online tutorials are available at http://www.structuralchemistry.org/pcsb/capp.php.

**Electronic supplementary material:**

The online version of this article (doi:10.1186/s13321-015-0079-1) contains supplementary material, which is available to authorized users.

## Background

Screening of organic small-molecule compounds has been a pivotal activity in the pharmaceutical industry as part of the drug discovery process. In the last decade, compound screening has increasingly been established and employed by academic laboratories due to many disease areas not being tackled by commercially oriented pharmaceutical industry, and also due to the availability of advanced technologies for the probing of biological systems [1].

The use of chemical tools and compound screening has therefore found new user clienteles, not all of whom are expert medicinal chemists and thus familiar with the properties of organic molecules. Recently, Baell and colleagues [2] highlighted a significant problem arising from the massively increased, non-expert compound screening in that molecules with promiscuous activities (pan-assay interference compounds, PAINs) are frequently being reported in the literature as (potential) hits in an undiscriminating fashion.

The concept of chemical spreadsheets is well established, and several different products have been developed in the past [3] that will store chemical data and present in a tabular form. Most such software is available from commercial providers, but there have also been freeware products, and increasingly web services provided by databases, such as ChemSpider [4] and the CCD Vault [5].

In the recent past, the concept of workflow has been implemented in many bio- and chemo-informatics approaches [6, 7]. Here, activities are classified into generic tasks that can be addressed by modular algorithms and thus combined by the end-user in a flexible fashion. Products in this category include the commercially available Pipeline Pilot (Accelrys, US) or InforSense (InforSense, UK). A freeware alternative is KNIME (Knime.com, Switzerland), based on the open source Eclipse platform, and CDK-Taverna [8] which builds on the Java libraries of the Chemistry Development Kit (CDK) [9].

Our own experience in collaborative work among medicinal chemistry, structural biology and biochemistry laboratories shows that data exchange, collection, archiving and publishing is very much done on a case-by-case basis, whereby simple tasks are often done repetitively and in many cases redundantly. Although the above spreadsheet or workflow software is able to deal with the requirements arising from drug screening projects in the academic setting, the actual deployment of such software by end-users is often hampered by access/availability, difficulty of installation and/or the perceived or real difficulty to learn how to use the software.

We set out to design a platform-independent Java application, based on our in-house developed collection PCSB [10], that should appeal to non-expert laboratories engaged in the handling of medium-sized compound libraries. Particular attention has been paid to making the learning and use of this software as convenient as possible. The portable Java application cApp enables the appraisal of compounds sourced from the commonly used formats of SMILES (simplified molecular-input line entry system; see specifications at [11]), InChI (International Chemical Identifier; see specification at [12]) and SDF (Structure Data Format; see Chemical Table File specification from December 2011 at [13]) files with respect to adherence to likeness rules. Compounds can also be input or manipulated via the embedded JChemPaint [14] chemical editor. Particular innovative features built into cApp are the identification of PAIN components in the appraised compounds, direct queries of the PubChem Compound Database [15] as well as similarity searches initiated with one mouse click.

## Implementation

cApp has been implemented in Java for maximum portability, capitalising on existing chemo- and bio-informatic Java libraries, namely the CDK [9], JChemPaint [14] and PCSB [10]. The data structure within cApp rests on the custom-programmed *Compound* object that handles all data relating to individual small-molecule compounds for this software. Access to the PubChem Compound Database is through the PubChem Power User Gate (PUG), which is an XML-based communication gateway to interrogate the database.

## Results

### Software features

**Figure 1:**
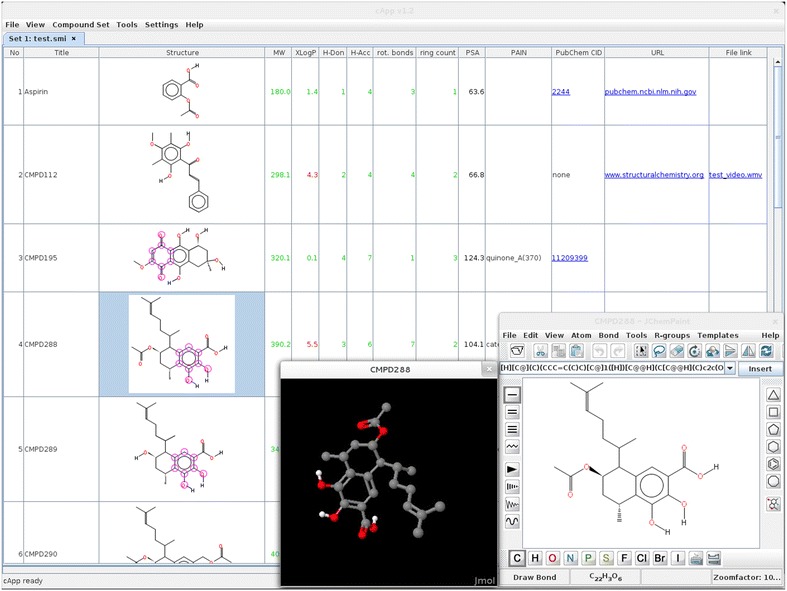
Screenshot of a project in the graphical user interface of cApp. Different tasks (appraisal, similarity searches) or sets of one project appear under different tabs. Views of a particular compound in the Jmol molecular viewer and the JChemPaint chemical editor are available with a single mouse-click. The project shown illustrates an appraisal task (loaded from the file ‘test.smi’). Views of CMPD288 in the Jmol molecular viewer as well as the JChemPaint chemical editor are activated.

cApp is a personal compound database software that allows the user to compare chemical descriptors and similarities of compounds, but also to annotate compound lists with their own data and information. A cApp project comprises all data and compound sets of a software session; a compound set is a particular list of compounds. In the GUI, a compound set is displayed as a table on a particular tab (see Figure 1). Automatically generated HTML, PDF and ASCII presentations of compound sets are identified by their set number. Conceptually, its functionality is divided up into tasks, presentation of results and convenience features. In the present version, the tasks of compound appraisal, similarity search and clustering can be performed. The compound appraisal task calculates physico-chemical properties and structural features, an analysis for compliance with various likeness criteria (drug-, lead- or fragment-like) [16] and the identification of PAINs components [17] using the SMSD maximum common subgraph (MCS) Tanimoto coefficient as criterion [18]. Similarity searches against user-provided libraries can be conducted using an MCS approach which builds on the CDK Fingerprint Tanimoto coefficient [18] or the PubChem Compound Database. For compound clustering, a MaxMin algorithm with subsequent k-Means clustering [19] has been implemented, based on the CDK Fingerprint Tanimoto coefficient as property. The user can annotate compounds with extra information by adding three types of data in additional columns containing either free text, a file link or a URL. Linked files and web content are available with a mouse click from the cApp GUI via the user’s preferred web browser.

The individual features of cApp are described in a detailed manual that is available together with the application (see also the Additional file 1). Online tutorials for typical scenarios have been prepared and can be accessed at the project web site.

### Assessment of similarity with pan-assay interference compounds (PAINs)

Baell and Holloway [17] have identified a set of chemical substructures that are frequently observed as effectors in compound screening and thus deemed to be promiscuous. In the compound appraisal task, cApp conducts SMARTS queries using 480 PAINs substructure filters that have been translated from the original rules in Sybyl Line Notation (sln) by Dr Rajarshi Guha (http://blog.rguha.net/?p=850). This conversion of the PAINs substructure filters from sln to SMARTS does not reproduce the original rules perfectly. For the present version of cApp, we have combined the three filters sets obtained from [20] into one set (pains.smt).

We have subjected a library of 50,000 compounds from the ChemBridge catalogue to PAINs filtering using the same SMARTS filters in cApp and PipelinePilot [21]. We also compared the results of PAINs-filtering in cApp with those obtained by the original sln rules. The results from this benchmarking indicate that there are small variations in the queries conducted by different software (see Table 1).

**Table 1:** Comparison of PAINs identification by different software/methodologies using a library of 50,000 compounds from the ChemBridge catalogue

Software	cApp v1.2	Sybyl	Matching entries
Rules	pains.smt [20]	sln [17]	
No of PAINs	5,790	6,001	5,788
Hits identified only in one approach	2	213	

Software	cApp v1.2	Pipeline Pilot	Matching entries
Rules	pains.smt [20]	pains.smt [20]	
No of PAINs	5,790	5,994	5,782
Hits identified only in one approach	8	212	

## Conclusions

With cApp, we have developed a personal, small-molecule database management software that should appeal to the non-expert user due to its ease of installation, intuitive handling and convenient execution of tasks. In future versions, we plan to include additional functionality, such as identification of duplicate entries, and direct query capability of further public compound repositories, such as ChEMBL and others.

## Availability and requirements

Project name: cApp.

Project home page: http://www.structuralchemistry.org/pcsb/capp.php.

Operating system(s): Platform independent.

Programming language: Java.

Other requirements: Java 1.7 or higher.

License: GNU AGPL v3.

Any restrictions to use by non-academics: None.

## Additional file

**Additional file 1: **The software manual accompanies this paper as supplementary information.
