# Supplementary material for: A practical Java tool for small-molecule compound appraisal
Source: J Cheminform. 2015 Jun 16;7:28. doi: 10.1186/s13321-015-0079-1 (PMC4469258; doi:10.1186/s13321-015-0079-1)
Supplement: Supplementary file 1 — Additional file 1: The software manual accompanies this paper as supplementary information. [file 13321_2015_79_MOESM1_ESM.pdf]

cApp v1.2

Table of Contents

1. SUMMARY......1
2. REQUIREMENTS......2
3. DESCRIPTION OF THE WORKING CONCEPT......2
3.1 Tasks and presentation of results......2
3.2 Likeness analysis......4
3.3 Similarity with pan-assay interference compounds (PAINs)......5
3.4 Similarity search......5
3.5 Compound clustering......6
3.6 Splitting libraries......7
3.7 Annotation......7
3.8 Convenience features......7
4. OPERATION......8
4.1 From the terminal......8
4.2 With the GUI......9
5. FILE FORMATS......9
6. DESCRIPTION OF THE GUI MENU BAR ITEMS......9
6.1 File......9
6.2 View......12
6.3 Compound Set......12
6.4 Tools......13
6.5 Settings......13
6.6 Help......13
6.7 Pop-up menu......14
7. NON-PCSB JAVA LIBRARIES USED IN THIS PROGRAM......15
8. REFERENCES......16
9. APPENDIX 1: COPYRIGHT, LICENCE AND DISCLAIMER......16
10. APPENDIX 2: EXAMPLES......18
10.1 Adding user-supplied data from an input file to a compound set......18
10.2 Performing a similarity search with a selected compound......18
10.3 Conversion of chemical data formats......19

1. Summary

cApp is a convenient and easy-to-use Java application that aids handling and storage of information about small-molecule compounds. With the application, the user can appraise compounds with respect to their physico-chemical properties and present structural information together with calculated or measured properties. Structures can be provided by the user in the form of SMILES, InChI, structure-data files (SDF) or added via the embedded chemical editor. The tasks performed by cApp include compound appraisal by calculation of common chemical descriptors and analysis with respect to adherence to likeness rules, as well as a substructure search for pan-assay interference components. The user can add data and annotation, and directly query the PubChem database. Similarity searches using a maximum common subgraph approach (Asad Rahman *et al.*, 2009) can be performed interactively or in terminal mode. cApp results are presented in a tabular view when using the software with its graphical user

interface. Results can also be written in HTML-format or exported as PDF. The output of data in ASCII format allows further processing of data with other suitable programs or scripts.
cApp uses the Chemistry Development Kit (Steinbeck *et al.*, 2006), an open-source Java library for chem- and bioinformatics, and associated software, JChemPaint (Krause *et al.*, 2000) as chemical editor, and routines developed within the Program Collection for Structural Biology and Biophysical Chemistry (Hofmann & Wlodawer, 2002). When using cApp, please cite XXX. Online tutorials are available at http://www.structuralchemistry.org/pcsb/capp.php.

2. Requirements

cApp requires JRE7 as the Java Runtime Environment. The software has been compiled and tested using Oracle Java 1.7.

3. Description of the working concept

This software is intended for work with small to medium-sized compound libraries. Especially when working with the GUI, please be mindful when loading extensive compound sets. The upper limit of compounds to be handled in one cApp session obviously depends on the resources of the computer system used. We have found that on machines with current typical specifications, sets with about 1000 compounds can still be reasonably well handled.
Therefore, the user will be prompted with a warning when attempting to read libraries that are larger than ~1000 compounds. Large library files in SDF format can be split using the split task. This task is not affected by large file sizes, as it does not create any cApp compound sets and only writes subsets to the disk.
Similarity searches that use large libraries can be carried out when starting the task from the terminal without the GUI (see section 3.4).

3.1 Tasks and presentation of results

The software has been conceived as an aid to handle and store information about small-molecule compounds in the context of drug discovery, screening and biological probing contexts. Conceptually, its functionality is divided up into

- tasks
- presentation of results, and
- convenience features.

Four tasks have been implemented:

- Compound appraisal: Calculation of physico-chemical properties and structural features, and analysis as to compliance with various likeness criteria and existence of PAINs components. User-provided data and annotation can be included and interactive convenience features are available.
- Similarity search: Libraries of small-molecule compounds can be queried using individual or multiple compounds for structural similarity with a maximum common subgraph approach. The PubChem Compound database can also be queried for similarity.
- Compound clustering: Using Tanimoto similarity, the compounds within one set are grouped into a user-specified number of clusters.
- Splitting of libraries (currently only for SDF libraries): Multi-compound files can be split into subsets with a user-specified number of entries each.

Projects and compound sets:

A project comprises all data and compound sets when running an instance of cApp. A compound set is a particular list of compounds. In the GUI, a compound set is displayed as a table on particular tab. Automatically generated HTML, PDF and ASCII presentations of compound sets are identified by their set number.

Presentation of results (using the GUI):
The GUI will be invoked when

- double-clicking the cApp jar-file (Windows)
- starting program without any switches from the command line
- starting program from the command line and including the `-gui` switch
- starting program from the command line via the `-load {...}` switch.

cApp uses tabbed panels to enable viewing of compound sets. For each compound set, a panel is added with the name of the set showing as label in the tab. For appraisal tasks, this panel shows a table comprising of twelve pre-set columns:

- ***No***, ***Title***, and ***Structure*** contain a running number, the compound ID, and the image of the 2-dimensional structure of the compound, respectively. 2D images of structures are rendered using the JChemPaint (Krause *et al.*, 2000) rendering features. Data in the ***Title*** column can be edited.
- The next seven columns contain information about the physico-chemical properties of the compounds: molecular mass (***M***), calculated logP (***clogP***), number of hydrogen bond donors (***H-Don***), number of hydrogen bond acceptors (***H-Acc***), number of rotatable bonds (***rot. bonds***), number of rings (***ring count***), and polar surface area (***PSA***). The colour mapping in these seven columns indicates the compliance with the selected likeness (***Drug like***, ***Lead like***, ***Fragment like***; see Table 3.2); green colour signals compliance, red colour shows violation and black colour indicates that this property is not part of the criteria set of the selected likeness. The type of likeness criteria selected is displayed as a tool tip for these column headers.
- The 11th column indicates whether a molecule contains an entity of a pan-assay interference compound (***PAIN***).
- In the 12th column (***PubChem CID***), a link to an entry for this compound in the PubChem Compound database is displayed, if this compound has been subjected to a PubChem search (see 6.7.2 , 6.7.4).
- Any information added by the user will be added on in columns 13 and higher. Columns with user-supplied data can be edited either by direct typing (number/text) or adding links through the pop-up menu (see 6.7.12 , 6.7.13).

(Tables for similarity search tasks are described in 3.4).
By clicking on the column header, the user can sort the table with respect to any tabulated criterion. All results can be saved either in HTML, PDF or ASCII format through the `File – Save results` functions.

Presentation of results (without the GUI):
The software can be invoked from the command line using the switches described in Table 4.1. If the `-gui` switch is not included, the requested task will be carried out without displaying the GUI. Results of all appraisal and similarity search tasks are then automatically reported in ASCII-formatted tables (no graphics included). These files are organised in an auto-generated directory called `capp_results_ascii`. If the `-html` switch is included, all results will also be written in HTML-formatted files which can be viewed in a web browser. The HTML files will be organised in the auto-generated directory `capp_results_html`.

3.2 Likeness analysis

Analysis of whether a compound has drug-like, lead-like or fragment-like properties is based on select chemical descriptors. cApp uses algorithms implemented in CDK to obtain values for the following descriptors:

**Property**	**Descriptor**	**Reference**
Molecular mass	WeightDescriptor	(Steinbeck *et al.*, 2006)
Lipophilicity: calculated logP	XLogPDescriptor	(Wang *et al.*, 1997; Wang *et al.*, 2000)
No of H-bond donors	HBondDonorCountDescriptor	(Steinbeck *et al.*, 2006)
No of H-bond acceptors	HBondAcceptorCountDescriptor	(Steinbeck *et al.*, 2006)
No of rotatable bonds	RotatableBondsCountDescriptor	(Steinbeck *et al.*, 2006)
No of rings	ConnectivityChecker	(Steinbeck *et al.*, 2006)
Polar surface area	TPSADescriptor	(Ertl *et al.*, 2000)

Table 3.1: Algorithms for CDK chemical descriptors used in cApp.

The criteria to be fulfilled if a compound can be classified as a drug-like, lead-like or fragment-like molecule have been described in the literature (see Table 3.2), most of which have been implemented in cApp.

**Property**	**Drug-like**	**Lead-like**	**Fragment-like**
	**Rule of 5**		**Rule of 3**
Molecular mass	≤ 500 Da	≤ 460 Da	≤ 300 Da
Lipophilicity: calculated logP	≤ 5	-4 ≤ clogP ≤ 4.2	≤ 3
No of H-bond donors	≤ 5	≤ 5	≤ 3
No of H-bond acceptors	≤ 10	≤ 9	n/a
No of rotatable bonds	n/a	≤ 10	≤ 3
No of rings	n/a	≤ 4	≤ 4
Polar surface area	n/a	n/a	≤ 60 $Å^2$
Aqueous solubility: logS*	n/a	≥ -5	n/a

*Table 3.2: Literature criteria for drug, lead and fragment likeness according to (Barker et al., 2008). *Not implemented in cApp.*

3.3 Similarity with pan-assay interference compounds (PAINs)

Baell and Holloway have identified a set of chemical substructures that are frequently observed as effectors in compound screening and thus deemed to be promiscous (Baell & Holloway, 2010). In the compound appraisal task, cApp conudcts SMARTS queries using 480 PAINs subtructure filters that have been translated from the original rules in Sybyl Line Notation (sln) by Rajarshi Guha (http://blog.rguha.net/?p=850). This conversion of the PAINs substructure filters from sln to SMARTS is not reproducing the original rules perfectly. For the present version of cApp, we have combined the three filters sets obtained from http://blog.rguha.net/?p=850 into one set (pains.smt). Additionally, there appear to be small variations in the queries conducted by different software.

We have subjected a library of 50,000 compounds from the ChemBridge catalogue to PAINs filtering using the same SMARTS filters in cApp and PipelinePilot (Pipeline Pilot, 2013). We also compared the results of PAINs filtering in cApp with those obtained by the original sln rules. See Table 3.3 for a summary.

**Software**	**cApp v1.2**	**Sybyl**	**Matching entries**
**Rules**	pains.smt (http://blog.rguha.net/?p=850)	sln (Baell & Holloway, 2010)	
**No of PAINs**	5790	6001	5788
**Hits identified only in one approach**	2	213	

**Software**	**cApp v1.2**	**PipelinePilot**	**Matching entries**
**Rules**	pains.smt (http://blog.rguha.net/?p=850)	pains.smt (http://blog.rguha.net/?p=850)	
**No of PAINs**	5790	5994	5782
**Hits identified only in one approach**	8	212	

Table 3.3: Comparison of PAINs identification by different software/methodologies using a library of 50,000 compounds from the ChemBridge catalogue.

3.4 Similarity search

Similarity searches within cApp to detect substructures (query) in a target molecule are performed using the maximum common subgraph approach implemented in SMSD (Small Molecule Subgraph Detector) (Asad Rahman *et al.*, 2009) and provided with the CDK libraries (class 'Isomorphism' with 'default' algorithm). The main parameter describing similarity between query and target molecule is the Tanimoto coefficient (Rogers & Tanimoto, 1960; Tanimoto, 1957). The Tanimoto coefficient is based on the cosine similarity between two n-dimensional vectors.

Two types of similarity search tasks can be performed with cApp:

- similarity search of a selected compound against a user-provided library in SDF format: here, all compounds of the library are processed the similarity search results will be added to the cApp project as a new compound set that appears as a tabbed panel

- similarity search of a selected compound against the PubChem Compound database: here, the user needs to provide a minimum Tanimoto coefficient (in percent) as well as a maximum number of hits to be accepted. based on these criteria, a PubChem similarity search is executed and the results downloaded at once. cApp will then process each compound obtained from the PubChem search in offline SMSD calculations and assess as to the similarity search parameters (see below).

The tabulated data reported in similarity searches are different from those of appraisal tasks. Results from similarity searches are presented as nine pre-set columns:

- ***No***, ***Title***, and ***Structure*** contain a running number, the compound ID, and the image of the 2-dimensional structure of the compound, respectively. Data in the ***Title*** column can be edited.
- ***Tanimoto similarity***: this reports the CDK Fingerprint Tanimoto coefficient (Asad Rahman *et al.*, 2009). Identical molecules have a Tanimoto coefficient of 1.
- ***Fragment size***: the number of fragments generated in the solution space, if the maximum common subgraph is removed from the target molecule. Amongst different solutions, those with lowest fragment sizes are preferred.
- ***Subgraph*** is ticked if the query molecule is a subgraph of the target molecule
- ***Stereo score***: a number which denotes the quality of the maximum common subgraph match. Higher stereo scores are preferred over lower scores.
- ***Stereo mismatch*** is ticked if the query and target molecules have different stereochemistry
- ***PubChem CID***: a link to an entry for this compound in the PubChem Compound database is displayed, if this compound has been subjected to a PubChem search (see 6.7.2 , 6.7.4).
- Any information added by the user will be added on in columns 11 and higher. Columns with user-supplied data can be edited either by direct typing (number/text) or adding links through the pop-up menu (see 6.7.12 , 6.7.13).

Note: when starting a similarity search from the terminal command line (`-smsd` switch), results will not be displayed in the GUI. The reason for this is that is possible to process a library of query molecules against a target library. As this (i) can take a very long time, and (ii) may result in a large number of compound sets to be displayed (as many as there molecules in the query compound set), results are not rendered in the GUI.

3.5 Compound clustering

Compounds in a particular set can be grouped into N clusters (Number to be provided by the user). The assessment criterion is a similarity measure, obtained by calculating the Tanimoto coefficient of each compound against all other compounds in the set (using the class 'Isomorphism' with algorithm 'mcsplus' which builds on the CDK Fingerprint Tanimoto coefficient (Asad Rahman *et al.*, 2009)). A matrix of pairwise similarity coefficients is then constructed with $sim_{ij} = (1 - Tanimoto_{ij})$. The first initial centre is chosen randomly, and the other initial (N-1) centres are then selected with a MaxMin algorithm (Gorse *et al.*, 1999) which selects the next centre as the most distant compound from the previously selected centres. Once all initial N centres have been chosen, the set is subjected to a k-Means clustering procedure which uses an ε-criterion for assessing convergence.

3.6 Splitting libraries

This task is intended for large library files (currently only available for libraries in SDF format) that need to be split into smaller subsets. The user needs to specify the number of entries in a subset. cApp will then process the provided library file and generate an individual file for each subset (numbered consecutively) until all compounds of the library have been processed. The last subset generated contains the remaining number of entries.

3.7 Annotation

Annotation of compounds in a set, either through automated cApp features or manually by the user, adds value to a set of compounds (database). This database is accessible and can be shared as cApp binary files, dissemination formats such as HTML and PDF, but also in formats that can be processed by computer software (ASCII files, SDF files).

Individual compounds can be annotated by the user by adding information in additional columns. Three types of data can be entered: ***Numbers/Text***, ***File link*** or ***URL***. Additional columns to existing compound sets may be added either by `Compound Set – Add column` or by `File – Add data column`. The first option adds an empty column and the user needs to specify the data type of that column. The second option reads a user-provided ASCII file with a list of compound IDs and particular information. When a compound ID matches an entry in the selected ***Compound Set*** in cApp, that set is updated by populating the new column with the given information.

The user can also select a cell in any of the additional columns with a `left-click` and then either enter (alpha)-numerical information or link a file or a web site, depending on the data type of the chosen column. File and web links need to be entered from the pop-up menu which can be obtained by `right-click`.

Any links will be presented through the user's web browser. cApp will attempt to determine the preferred web browser on the user's desktop. If it cannot obtain that information, the user will be prompted to enter the location of the web browser executable. Alternatively, the user can set the location of the web browser executable under `Settings`.

3.8 Convenience features

cApp is a personal compound database software that allows the user to compare chemical descriptors and similarities of compounds, but also annotate compound lists with their own data and information. The fact that the software can be run in interactive mode with a GUI, but also in batch mode from a terminal line, allows the usage of cApp as a tool to quickly generate compound information from any input format (SMILES, InChI, SDF) and thus, for example, convert easily from SMILES to InChI/SDF, etc. A list of compounds appraised in batch mode with either the `-png` or `-svg` switch will generate 2D structure images for all compounds.

Other convenience features include compound input by drawing in a chemical editor, the generation of 3D conformation from compounds provided as 2D information. It should be noted that this is for convenience only and cannot replace a rigorous QM/semi-empirical modelling study.

Data mining of public compound databases is an important component of modern cheminformatics, and cApp thus offers direct querying of the PubChem Compound database via the PubChem Power User Gate (PUG), which is an XML-based communication gateway to interrogate the database. A user-initiated PubChem query consists of several XML queries directed at the PUG. The individual XML queries are sent by cApp in 3.2 s intervals. cApp uses an internal time-out of 180 s for any PubChem query.

4. Operation

4.1 From the terminal

The program can be started from the terminal by:

```
java -jar {capp.jar file} [switches]
```

Switch	Function
`-appraise`	Runs the appraise task (default)
`-ascii`	Write results in ASCII format (directory: capp_results_ascii)
`-autoselect`	Auto-select largest entity when reading SDF or SMILES.
`-cluster {N}`	Group compounds into {N} clusters
`-debug`	Debug option
`-drug`	Evaluate for drug likeness (Lipinski's Rule of 5)
`-fragment`	Evaluate for fragment likeness (Rule of 3)
`-gui`	Start the program with the graphical user interface
`-h`	Print help
`-html`	Generate results in HTML format (directory: capp_results_html)
`-i {input file}`	Input file with compounds to process
`-inchi`	Input file contains InChI code
`-lead`	Evaluate for lead likeness
`-load`	Load a previously saved cApp project
`-maxsets {N}`	Maximum number of compound sets in the project (default: 10)
`-pdf`	Write results into PDF files (directory: capp_results_pdf)
`-pdf landscape`	PDF paper orientation is Landscape (default).
`-pdf portrait`	PDF paper orientation is Portrait.
`-pdf bondlength`	Structure images are drawn with same bond length (default).
`-pdf fixed`	Structure images have the same fixed size.
`-png`	Write PNG images of compounds (directory: capp_images)
`-pubchem`	Search for entry in PubChem when conducting the appraise task
`-sdf`	Input file is an SDF file
`-smi`	Input file contains SMILES code
`-smsd {library file}`	Similarity search of {input file} against {library file} in SDF format
`-split {N}`	Split an SDF library into subsets of {N} entries each.
`-svg`	Write SVG images of compounds (directory: capp_images)
`-3d`	Attempt to generate 3D coordinates

Table 4.1: Program options from the terminal (switches).

4.2 With the GUI

The GUI menu bar and pop-up menu items are described below (6.). In the GUI, individual compound sets appear as a table on separate tabbed panels. Each table consists of a number of pre-set columns (different for appraise and similarity search tasks); user-provided data for each compound can be added to columns following the pre-set columns. Indvidual columns can not be moved, but hidden using the `View – Column display` feature. These view settings also apply to the HTML-formatted output. Tables can be re-sorted as per a chosen criterion (column) by clicking on the column header.
In order to select a compound, any column in the row listing the compound can be selected by a `left-click`. Multiple rows can be selected by `Ctrl + left click`.

5. File formats

Information about the chemical molecular file formats can be found on the web:

Format	Web site
Structure-data file (SDF)	http://en.wikipedia.org/wiki/Chemical_table_file
Simplified molecular-input line-entry system (SMILES)	http://en.wikipedia.org/wiki/Simplified_molecular-input_line-entry_system
International Chemical Identifier (InChI)	http://en.wikipedia.org/wiki/International_Chemical_Identifier<br>http://www.iupac.org/inchi/

Table 5.1: Chemical molecular file formats and links to web sites describing their characteristics.

6. Description of the GUI menu bar items

6.1 File

6.1.1 Load project

With this option, previously saved cApp projects can be loaded.

6.1.2 Save project

With this option, the present project can be saved as a cApp binary data file.

6.1.3 Add compounds as new set

This function reads a user-provided ASCII file, establishes a new compound set in cApp and populates it with the compounds imported as SMILES or InChI codes, or from SD-formatted files. Files that contain multiple compounds as SMILES codes need to have one compound per line, with the SMILES code and compound ID separated by space(s), e.g.:

```
Oc2ccc(C=Cc1cc(O)cc(O)c1)cc2 Resveratrol
c1c(c(ccc1O)/C=C\1/C(=O)N(C(=S)S1)CC(=O)Nc1ccc(cc1)Oc1ccc(cc1)Cl)O Catechol rhodanine
...
```

Files that contain InChI codes need to have the following format:

```
InChI=1S/C14H12O3/c15-12-5-3-10(4-6-12)1-2-11-7-13(16)9-14(17)8-11/h1-9,15-17H Resveratrol
InChI=1S/C24H17ClN2O5S2/c25-15-2-7-18(8-3-15)32-19-9-4-16(5-10-19)26-22(30)13-27-23(31)21(34-
24(27)33)11-14-1-6-17(28)12-20(14)29/h1-12,28-29H,13H2,(H,26,30) Catechol rhodanine
...
```

It is advisable to always provide compound names when adding compounds to cApp; if no names are provided, the program will label these entries as `unnamed_{no}`.
If an SDF file is imported that possesses data in the SDF property block, the user will be presented with a dialog to choose which of those should be added to the appraise table as user-defined data (default: none).

6.1.4 Add compounds to selected set

With this function, a user-provided ASCII file with compounds in either `SMILES`, `InChI` or `SDF` format is read. The compounds are added to the selected compound set. The selected compound set is the one currently showing as table in the GUI. The new compounds are added at the end of the presently selected set.
If import from an SDF file is chosen, and the file contains SDF properties with a key that matches a column name in the user-defined data section, then that SDF property willbe automatically added in the user-defined column. If an SDF property with the key PubChem CID is present, the value will be automatically added to the ***PubChem CID*** column.

6.1.5 Add data column

In this feature, the user can specify a two column ASCII file that contains data for compounds in the presently active tab. A new column is added at the end of the table of the presently active tab and populated with information from the user-provided ASCII file, if a compound ID in the ASCII file matches the compound ID in the presently active tab.
When processing the input file, cApp determines whether the data to be added are of the type (alpha)-numerical, file link or web link (URL). In order for file links to be recognised, the files must exist in the specified location.

6.1.6 Split SDF

Large SDF libraries can be split into smaller subsets using this feature. The user needs to provide the location of the SDF library to be processed, the output directory for the SDF files containing the subsets and the number of entries per subset.

6.1.7 Write SDF

With this option, compound sets can be saved in the SD file format. One can chose to save either `The active set`, `All sets into separate files` or `All sets into one file`. For `The active set`, the user can select a file name, for the other options, only a directory to write into needs to be given. If `All sets into separate files` is chosen, the individual files will be organised into separate directories called `set_1`, `set_2`, etc.

6.1.8 Write SMILES

With this option, compound sets can be saved in SMILES format. One can chose to save either `The active set`, `All sets into separate files` or `All sets into one file`. For `The active set`, the user can select a file name, for the other options, only a directory to write into needs to be given. If `All sets into separate files` is chosen, the individual files will be organised into separate directories called `set_1`, `set_2`, etc. File names are assigned automatically following the format `set_{set number}_????.png`. The 4-digit running number is the number shown in the first column of each compound set.

6.1.9 Write InChI

With this option, the compounds can be saved in InChI format. One can chose to save either `The active set`, `All sets into separate files` or `All sets into one file`. With `The active set`, just the compounds of the active compound set are saved and the user can select a file name. For `All sets into separate files,` only a directory to write into needs to be given and the individual files will be organised into separate directories called `set_1`, `set_2`, etc. With `All sets into one file`, all compounds of the project can be saved. File names are assigned automatically following the format `set_{set number}_????.png`. The 4-digit running number is the number shown in the first column of each compound set.

6.1.10 Write InChI Key

InChI keys are often used for web searches and can also be saved. The procedure is the same as described above under 6.1.9 .

6.1.11 Write structure images

This feature writes 2D structure images in `PNG` or `SVG` format of compounds either for `The active set` or `All sets`. File names are assigned automatically following the format `set_{set number}_????.{png/svg}`. The 4-digit running number is the number shown in the first column of each compound set. If `The active set` is chosen, the user needs to specific a directory for the image files. If `All sets` is chosen, the image files will be automatically organised into separate directories called `set_1`, `set_2`, etc in the user-specified location.

6.1.12 Save results as PDF

This feature generates a single PDF file of the results in either `The active set` or `All sets`. Options that can be selected include paper orientation, as well as structure images drawn with the same bond lengths or in fixed overall size.

6.1.13 Save results in HTML format

With this option, results can be saved as HTML-formatted files. This can either be done for `The active set` or `All sets`. The latter option will save all current compound sets at once. Embedded images and accompanying SMILES or SD files will be organised into separate directories (called `files_1`, `files_2`, etc) for each set. The user can select a directory to write into; files are automatically named by cApp. If `All sets` is chosen, an index file (`index.htm`) will be generated that serves as a overview of contents for this project.

6.1.14 Save results in ASCII format

With this option, results can be saved as ASCII files. This can either be done for `The active set` or `All sets`. The latter option will save all current compound sets at once. The user can select a directory to write into; files are automatically named by cApp.

6.1.15 Exit

This exits the program after warning the user that unsaved changes may be lost.

6.2 View

6.2.1 Column display

Here, individual columns can be selected to be shown or hidden. The same settings will be applied to the HTML-formatted output.

6.3 Compound Set

6.3.1 Project description

With this option, a `Project Title` and `Project Description` can be can be set for the current project. The 'project' comprises all tabbed panels in the session.

6.3.2 Compound set description

With this option, a `Compound Set Title` and `Compound Set Description` can be can be set for the current active compound set. By default, the title is the file name of the loaded set, and the description describes the task (appraisal, SMSD) that has been carried out for this set.

6.3.3 Add compound

With this option a new compound can be added to the end of the current list. The ***SMILES*** or ***InChl*** code has to be entered manually. Alternatively, a structure can be drawn using the embedded version of JChemPaint as ***Chemical Editor***.
If cApp is started without any compound set, a new set will automatically be established. Otherwise, the new compound will be added to the currently active compound set.

6.3.4 Remove selected compounds

The compounds selected in the currently active set will be removed.

6.3.5 Add column

With this option, an empty column will be added at the end of the table. The user can attribute a header to the column, and also needs to specify the type of data to be entered into this column. Data can be of the type `Numbers/Text`, `File link` or `URL`.

6.3.6 Image parameters

When using `Image parameters` from the menu bar, the settings are applied to all compounds of the active set. If the parameters for an individual compound are to be adjusted, the feature ***Image parameters*** from the pop-up menu (6.7.6) can be used.
The ***Bond length*** parameter specifies the desired bond length of the rendered compound in pixels.
2D images are rendered to fill a rectangle defined by ***Image Width*** and ***Image Height***. By default, the image scale, and thus width and height, are automatically calculated (***Auto Image Scale*** is set to `Yes`). If ***Auto Image Scale*** is set to `No`, the user-defined ***Image Width*** and ***Image Height*** parameters are applied.
Implicit hydrogens can be shown or hidden by setting ***Render with Hydrogens*** accordingly.

6.3.7 Close compound set

This option closes the active compound set after displaying a confirmation dialog.

6.4 Tools

6.4.1 Likeness analysis

This option allows the user to change the type of likeness (Lipinski's Rule of 5, lead-like or fragment-like aka Rule of 3) criteria to be applied when appraising the chemical descriptors of compounds in the compound set. If the criteria for the selected likeness are met, the values are highlighted green; if they are not met, the values are shown in red. If they are black, the property is not part of the chosen rule set.

6.4.2 Identical entries in PubChem

With this feature, one can search for identical entries in the PubChem compound database, either `For active set` or `For selected compounds`. The search process can be stopped by clicking the `Stop PubChem query` button above the tabbed panel.

If identical entries are found in the database, the compound identifier (CID) link to the first found entry will be added in in the ***PubChem CID*** column. If no entries are found, the entry `none` is added to indicate that this compound has been searched for.

6.4.3 Cluster compounds

The currently active compound set is subjected to a clustering ***based on Tanimoto similarity***. The user is prompted for the number of clusters to be established.

6.4.4 Generate 3D coordinates

This feature uses CDK's ModelBuilder3D with its default force field to generate a 3D conformation of molecules in the selected compound set. One can chose to generate 3D coordinates either `For active set` or `For selected compounds`. The calculation will be done in the background as indicated by the progress bar. The user can abort the calculation by clicking the `Stop 3D coordinates generation` button above the tabbed panel.

The selected compound set is updated with the new coordinates. In order to access the results the user needs to write out compound coordinates in SD format (`File – Write SDF`). There is no obvious change visible on the cApp panel the 3D confromation can be inspected with the molecular viewer (select a cell in the table by `left-click` to open the pop-up menu, and then select `Open in molecular viewer`). Caveat: The CDK ModelBuilder3D achieves reasonable geometry for some but not all molecules. Further, there is no guarantee that the stereochemistry is correctly considered.

6.5 Settings

6.5.1 Import settings

If choosing `Auto-select`, cApp will try to find the largest connected set of atoms when importing compounds provided in SDF or SMILES format.

6.5.2 Local settings

Here, one can define local settings, such as the location of the web browser executable and the highlight colour for substructure identification.

6.6 Help

6.6.1 About

Displays the program version.

6.7 Pop-up menu
A pop-up menu appears when the user selects a cell in the table by `left-click` and then performs a `right-click`. Select options from the ones described below will be presented, depending on which column the highlighted cell belongs to.

6.7.1 Remove compound from set
This function removes the highlighted compound from the compound set.

6.7.2 Find PubChem entry
With this feature, one can search for identical entries in the PubChem compound database for the selected compound. If identical entries are found in the database, the compound identifier (CID) link to the first found entry will be added in in the ***PubChem CID*** column. If no entries are found, the entry `none` is added to indicate that this compound has been searched for.

6.7.3 Edit PubChem entry
If for some reason the ***PubChem CID*** needs to be changed, this can be done with this feature. A dialog window will open where the user can provide a PubChem CID.

6.7.4 Similarity search in PubChem
With this feature, one can search for similar entries in PubChem for the selected compound. The user will be asked to provide the minimum `Tanimoto Threshold (%)` and the `Max. number of results`. The similarity search will start and a new tab will be added containing the compound set retrieved from PubChem. The new compound set will be updated with similarity search parameters for each compound continuously until done.

6.7.5 Similarity search in a library
This feature processes a user-provided library (in SD format) in an SMSD similarity search against a selected compound. The user will be asked to choose a library in SDF format to be processed. The similarity search will start and a new tab will be added containing the compound set of the library. The table in the new set will be updated continuously until the search is done.

6.7.6 Image parameters
Here, the image rendering parameters can be changed for the selected compound. The options are as described under 6.3.6 .

6.7.7 Save image
This feature writes 2D structure images in `PNG` or `SVG` format for the selected compound. The file name is assigned automatically following the format `{Compound title}.{png/svg}`.

6.7.8 Generate 3D coordinates
This feature uses CDK's ModelBuilder3D with its default force field to generate a 3D conformation of selected molecule. The calculation will be done in the background as indicated by the progress bar. In order to access the results the user needs to write out compound coordinates in SD format (`File – Write SDF`). There is no obvious change visible on the cApp panel but the generated structure can be inspected with the molecular viewer by selecting a cell in the table by `left-click` to open the pop-up menu, and then select `Open in molecular viewer`). Caveat: The CDK ModelBuilder3D achieves reasonable

geometry for some but not all molecules. Further, there is no guarantee that the stereochemistry is correctly considered.

6.7.9 Update 3D coordinates from external SDF file

A three-dimensional conformation for the selected compound can be imported from a user-provided SDF file. This feature does not affect any other properties of the compound and just updates the atomic coordinates stored for this compound in the SDF component of the entry. When loading an external SDF file, cApp will perform a check to validate that the imported molecule matches the compound entry. For this check, the unique SMILES codes of compound entry and new molecule are compared.

6.7.10 Open in molecular viewer

This opens the selected compound in a Jmol (Steinbeck *et al.*, 2003) molecular viewer window.

6.7.11 Open in chemical editor

This opens the selected compound in the embedded version of JChemPaint (Krause *et al.*, 2000). When closing the JChemPaint session with ***Accept***, the selected compound entry will be updated with the modified structure; note that this will result in a 2D structure.

6.7.12 Add file link

With this feature, the user can add a file link in a user-defined column of the type `File link`. In order for file links to be recognised, the files must exist in the specified location.

6.7.13 Add URL

With this feature, the user can add a URL in a user-defined column of the type `URL`. when navigating to the URL, the user will be asked to provide a path to the executable of the web browser. The defined path can also be edited through `Setting – Local settings`.

6.7.14 Remove column

This option allows for removal of the selected column.

6.7.15 Show meta data

This option will display a table that presents the meta data available for the selected compound. Meta data are resourced from tags (if present) in the SD-formatted file. In any case, the compound title, InChI Code, Key and Aux information, as well as the SMILES code will be presented.

7. Non-PCSB Java libraries used in this program

cApp makes use of the following Java libraries not authored by us:

- CDK (version 1.4.19) , JChemPaint (version 3.3-1210), Jmol (version 12.0) and JNI-InChI are distributed with the GNU Lesser General Public License.
- Apache Commons are distributed with the following license: http://www.apache.org/licenses/LICENSE-2.0.
- iText is distributed under the Affero General Public License (AGPL).
- Apache Batik SVG toolkit

The k-Means algorithm implemented in cApp is an adaptation of code originally written by Selcuk Orhan Demirel and obtained from GitHub.

8. References

Asad Rahman S., Bashton M., Holliday G.L., Schrader R. & Thornton J.M. (2009) Small Molecule Subgraph Detector (SMSD) Toolkit. *J. Cheminformatics* **1**, 12.
Baell J.B. & Holloway G.A. (2010) New substructure filters for removal of pan assay interference compounds (PAINS) from screening libraries and for their exclusion in bioassays. *J. Med. Chem.* **53**, 2719-2740.
Barker J., Hesterkamp T. & Whittaker M. (2008) Integrating HTS and fragment-based drug discovery. *Drug Discovery World* **Summer 2008**, 69-75.
Ertl P., Rohde B. & Selzer P. (2000) Fast Calculation of Molecular Polar Surface Area as a Sum of Fragment-Based Contributions and Its Application to the Prediction of Drug Transport Properties. *J. Med. Chem.* **43**, 3714-3717.
Gorse D., Rees A., Kaczorek M. & Lahana R. (1999) Molecular diversity and its analysis. *Drug Discovery Today* **4**, 257-264.
Hofmann A. & Wlodawer A. (2002) PCSB - a program collection for structural biology and biophysical chemistry. *Bioinformatics* **18**, 209-210.
Krause S., Willighagen E. & Steinbeck C. (2000) JChemPaint - Using the Collaborative Forces of the Internet to Develop a Free Editor for 2D Chemical Structures. *Molecules* **5**, 93-98.
Pipeline Pilot (2013) Pipeline Pilot V9.1 (2013) BIOVIA, Dassault Systèmes, San Diego, California. , .
Rogers D.J. & Tanimoto T.T. (1960) A Computer Program for Classifying Plants. *Science* **132**, 1115-1118.
Steinbeck C., Han Y., Kuhn S., Horlacher O., Luttmann E. & Willighagen E. (2003) The Chemistry Development Kit (CDK): An Open-Source Java Library for Chemo- and Bioinformatics. *Journal of Chemical Information and Computer Sciences* **43**, 493-500.
Steinbeck C., Hoppe C., Kuhn S., Floris M., Guha R. & Willighagen E.L. (2006) Recent developments of the chemistry development kit (CDK) — an open-source java library for chemo- and bioinformatics. *Curr. Pharm. Des.* **12**, 2111-2120.
Tanimoto T.T. (1957) . *IBM Internal Report* **17th Nov. 1957**, .
Wang R., Fu Y. & Lai L. (1997) A New Atom-Additive Method for Calculating Partition Coefficients. *J. Chem. Inf. Comp. Sci.* **37**, 615-621.
Wang R., Gao Y. & Lai L. (2000) Calculating partition coefficient by atom-additive method. *Persp. Drug Discov. Des.* **19**, 47-66.

9. Appendix 1: Copyright, Licence and Disclaimer

Copyright © 1999-2015
Hofmann Laboratory, Structural Chemistry Program
Eskitis Institute, Griffith University.
http://www.structuralchemistry.org/pcsb/

Licence
This program is free software; you can redistribute it and/or modify it under the terms of the GNU Affero General Public License version 3 as published by the Free Software Foundation. You should have received a copy of the GNU Affero General Public License along with this program (file COPYING); if not, see http://www.gnu.org/licenses or write to the Free Software Foundation, Inc., 51 Franklin Street, Fifth Floor, Boston, MA, 02110-1301 USA.

Disclaimer

This program is distributed in the hope that it will be useful, but without any warranty; without even the implied warranty of merchantibility or fitness for a particular purpose. See the GNU Affero General Public License for more details.

10. Appendix 2: Examples

10.1 Adding user-supplied data from an input file to a compound set
Start the program with the GUI

```
java -jar {capp_jar_file} -gui
```

and establish a new compound set using the provided `test.smi` example by using `File – Add compounds as new set – SMILES`. A new tab is added and labelled ***Set 1: test.smi***.

The name of this compound set can be changed with the feature `Compound Set – Compound set description`.

Then use `File – Add data column` and load the provided example `test_dsf_data.txt`. Provide a name for the new column (e.g. ***DSF***). Information will then be added to those compounds where a match between the compound ID in the file `test_dsf_data.txt` and the compound set `test.smi` has been found.

Data can be supplied in ASCII input files consisting of two columns; in one column, the compound ID is to be given, in the other column the data values are to be given. After reading the file, cApp determines whether the data to be added is (alpha)-numerical, a file link or a web link (URL). In order for file links to be recognised, the files must exist in the specified location.

The provided example file `test_video_data.txt` shows how to define list of file links. Note that the syntax will differ, depending on the operating system:

Linux
```
CMPD337 /home/pcsb/capp/examples/test_video1.wmv
CMPD303 /home/pcsb/capp/examples/test_video2.wmv
CMPD288 /home/pcsb/capp/examples/test_video3.wmv
CMPD112 /home/pcsb/capp/examples/test_video4.wmv
```

Mac OSX
```
CMPD337 /Mac HD/Documents/capp/examples/test_video1.wmv
CMPD303 /Mac HD/Documents/capp/examples/test_video2.wmv
CMPD288 /Mac HD/Documents/capp/examples/test_video3.wmv
CMPD112 /Mac HD/Documents/capp/examples/test_video4.wmv
```

Windows
```
CMPD337 C:data\capp\examples\test_video1.wmv
CMPD303 C:data\capp\examples\test_video2.wmv
CMPD288 C:data\capp\examples\test_video3.wmv
CMPD112 C:data\capp\examples\test_video4.wmv
```

10.2 Performing a similarity search with a selected compound
Start the program with the GUI

```
java -jar {capp_jar_file} -gui
```

and establish a new compound set using the provided `test.smi` example by using `File – Add compounds as new set – SMILES`. A new tab is added and labelled ***Set 1: test.smi***.

Select a compound by a `left-click` on any cell in the row of the desired compound. Then `right-click` to obtain a pop-up menu. Select `Similarity search` and use the provided file `test.sdf` as a library to be searched.
The similarity search will start and a new tab will be added containing the compound set of the library. The table in the new set will be updated continuously until the search is done. In the above example, the compound selected as query molecule will be found with a Tanimoto similarity of 1.0.

10.3 Conversion of chemical data formats

Compounds can be read into cApp from either SMILES, InChI or SDF information. When running the software with the GUI, compound sets can be conveniently exported as any of those formats using the `File – Write` features.

In terminal mode (without the GUI), compound sets are easily converted into all chemical data formats that are automatically generated when running an appraise task with HTML output. For example, to convert a set of compounds provided as SMILES codes, the command

```
java -jar {capp_jar_file} -i test.smi -html
```

will appraise the compound set in test.smi and write SMILES, InChI, InChI Key and SDF files for all individual compounds as well as multi-compound files with those formats to the directory `capp_results_html/files_1`.
